# Disruption of Inferior Longitudinal Fasciculus Microstructure in Parkinson's Disease: A Systematic Review of Diffusion Tensor Imaging Studies

**DOI:** 10.3389/fneur.2018.00598

**Published:** 2018-07-26

**Authors:** Maryam Haghshomar, Mahsa Dolatshahi, Farzaneh Ghazi Sherbaf, Hossein Sanjari Moghaddam, Mehdi Shirin Shandiz, Mohammad Hadi Aarabi

**Affiliations:** ^1^Faculty of Medicine, Tehran University of Medical Sciences, Tehran, Iran; ^2^Department of Medical Physics, Zahedan University of Medical Sciences, Zahedan, Iran

**Keywords:** diffusion tensor imaging (DTI), inferior longitudinal fasciculus (ILF), Parkinson's disease, white matter microstructure, fractional anisotropy (FA), mean diffusivity (MD)

## Abstract

Parkinson's disease (PD) is a neurodegenerative disorder accompanied by a series of pathological mechanisms which contribute to a variety of motor and non-motor symptoms. Recently, there has been an increasing interest in structural diffusion tensor imaging (DTI) in PD which has shed light on our understanding of structural abnormalities underlying PD symptoms or its associations with pathological mechanisms. One of the white matter tracts shown to be disrupted in PD with a possible contribution to some PD symptoms is the inferior longitudinal fasciculus (ILF). On the whole, lower ILF integrity contributes to thought disorders, impaired visual emotions, cognitive impairments such as semantic fluency deficits, and mood disorders. This review outlines the microstructural changes in ILF associated with systemic inflammation and various PD symptoms like cognitive decline, facial emotion recognition deficit, depression, color discrimination deficit, olfactory dysfunction, and tremor genesis. However, few studies have investigated DTI correlates of each symptom and larger studies with standardized imaging protocols are required to extend these preliminary findings and lead to more promising results.

## Introduction

Parkinson's disease (PD), the second most prevalent neurodegenerative disorder, is accompanied by several motor (including muscular rigidity, bradykinesia, and resting tremor) (1, 2) and non-motor symptoms (3). Accumulation of proteins such as α-synuclein has been recognized as the main pathological mechanism in PD, which adversely affects synaptic function (4). As longitudinal studies have shown, Lewy body accumulation in PD arises from the midbrain and progresses to the limbic, frontal, parietal, followed by the temporal and occipital regions; this pattern of progression supports the prion theory in PD (5, 6). In addition, concomitant Alzheimer's disease pathology, i.e., neurofibrillary tangles (NFT) which consist of tau proteins, and plaques of beta-amyloid is a common observation in patients who have developed PD with dementia. NFTs, especially, can lead to axonal damage and contribute to white matter microstructural (WM) changes in different brain regions and tracts, which are consistently reported in patients with PD (7–10).

Importantly, various studies have shown disruptions in the occipital and temporal regions in association with motor and non-motor symptoms. For example, immunoprecipitation studies have confirmed reduced neurogranin levels, in addition to the decreased interaction between α-synuclein and neurogranin in the temporal cortex in patients suffering from PD (11). Likewise, patients with PD have shown significant differences in the gray matter (GM) volume (12), and in WM underlying the temporal, and occipital cortex as compared with normal controls (13, 14). In addition, recent studies have shown altered functional and structural connectivity in occipital and temporal lobes in PD and in association with its symptoms (15, 16).

In this context, diffusion tensor imaging (DTI) is a novel approach that has revolutionized our understanding of white matter microstructural abnormalities, and their correlation with clinical manifestations in PD (10). This method measures different parameters regarding the diffusion of water molecules on the micron scale and provides information about tissue integrity and direction of WM fibers within a voxel, based on the diffusion restriction caused by local environment structure including cell membranes, myelin sheath, organelles or even macromolecules (17).

The fractional anisotropy (FA) index is the most widely used DTI parameter for measuring the degree of anisotropy in the diffusion of water molecules in tissue, which represents the brain microstructural coherence and maturity (18, 19). The degree of anisotropy mostly depends on the number and density of axons and decreases in neurodegeneration (20). The mean diffusivity (MD), however, is direction-independent and reflects the total amount of diffusivity in a voxel. Freedom of water diffusion and lower tissue density contribute to higher MD. Two other diffusivity measures are the axial diffusivity (AD) parallel to the WM fascicles and the radial diffusivity (RD) perpendicular to them. Altered AD values reflect axonal damage and fragmentation, whereas changes to the RD values are representative of axonal density and diameter, and myelination.

There are three main techniques of DTI imaging analysis: (1) Region of interest (ROI) analysis extracts voxel values of specific regions of interest, previously assigned either manually and thus dependent on the operator, or via automated regional segmentation, in which improper spatial normalization can impose a major limitation (21, 22). (2) Voxel-based analysis (VBA) is an automated method which compares diffusion metrics in every voxel of the whole brain normalized to a template. In this method, the analysis is susceptible to alignment inaccuracy. In addition, images are smoothed, but there is no standard for the extent to which images are smoothed. In contrast to the limited number of comparisons in ROI, multiple comparisons in VBA can decrease the statistical power (23, 24). Tract-Based Spatial Statistics (TBSS) is a form of VBA, in which each individual's FA is projected to the FA skeleton. Limited amount of WM in a skeleton leads to higher sensitivity by reducing registration error and partial volume effects (23). (3) Tractography or fiber tracking is a semi-automated modeling technique used to reconstruct neuronal tracts passing through voxels with similar FA or MD, possibly indicating similar pathology. It does not require smoothing but is partly manual and is not able to search whole brain (23, 25–28).

Visual categorization and recognition initiate from the primary visual cortex, located in the occipital lobe, continues via a series of coordinated visual areas and eventually reaches the ventral temporal cortex. Inferior Longitudinal Fasciculus (ILF) is the main associative fiber tract which connects occipital and ventro-anterior temporal lobes (29, 30). More precisely, the anterior part connects the posterior occipitotemporal region, including the fusiform gyrus and the visual word form area, to the temporal lobe. The posterior part connects the inferior occipital gyrus to the posterior occipito-temporal region (31). Anatomically, the ILF is divided into three subparts based on the occipital terminations: the fusiform, the lingual and the dorsolateral-occipital (32). Functions attributed to the ILF consist of the object and facial visual recognition, intellectual abilities and emotional regulations. All these functions stem from the ventral (semantic) visual pathway that fast and directly connects V1 and V2 visual areas to the inferior temporal lobe (33, 34).

Numerous neuropsychological syndromes have been imputed to the disruption of specific fiber connections between occipital and temporal cortices, and most importantly the ILF (34). Deficits in ILF integrity can result in an inability to name objects (35). Reduced ILF FA index, is observed in adolescent patients with schizophrenia with visual hallucinations (36). ILF has manifested microstructural changes in dementia with Lewy bodies (37). There is also evidence of ILF changes in autism spectrum disorder (38).

In the present study, in order to provide an informative outlook of the specific WM fiber tract disruptions in PD and their associations with symptomatology and pathology, we aimed to review microstructural alterations of ILF integrity in patients with PD.

### Search strategy and data extraction

We performed a systematic search of the published literature to identify the studies that investigated the involvement of ILF disruptions associated with PD pathology and symptomatology using DTI. We used the broad search terms: “Diffusion Tensor imaging OR Diffusion tensor MRI OR Diffusion MRI OR DTI” AND “Parkinson's disease OR Parkinson disease OR PD.” We searched electronic databases including Embace, Scopus, and PubMed from 1980 to May 2018. This was complemented by manual searching of the related papers through the list of references. To avoid duplication, the results were imported to Covidence software and articles were separately screened by two of the authors (M.H. and M.H.A.). In case of disagreement, a third person (M.D.) interfered to decide whether to include or exclude articles. Among the search results, abstracts were screened for relevance. Studies which had investigated diseases other than PD, or had used imaging methods other than DTI were excluded. Full papers were obtained for studies published in English that performed DTI in PD group, and further assessed if they had investigated whole WM microstructure or particularly assessed one or more WM tracts including ILF in PD patients to be included in this systematic review.

## Results

**Figure 1:**
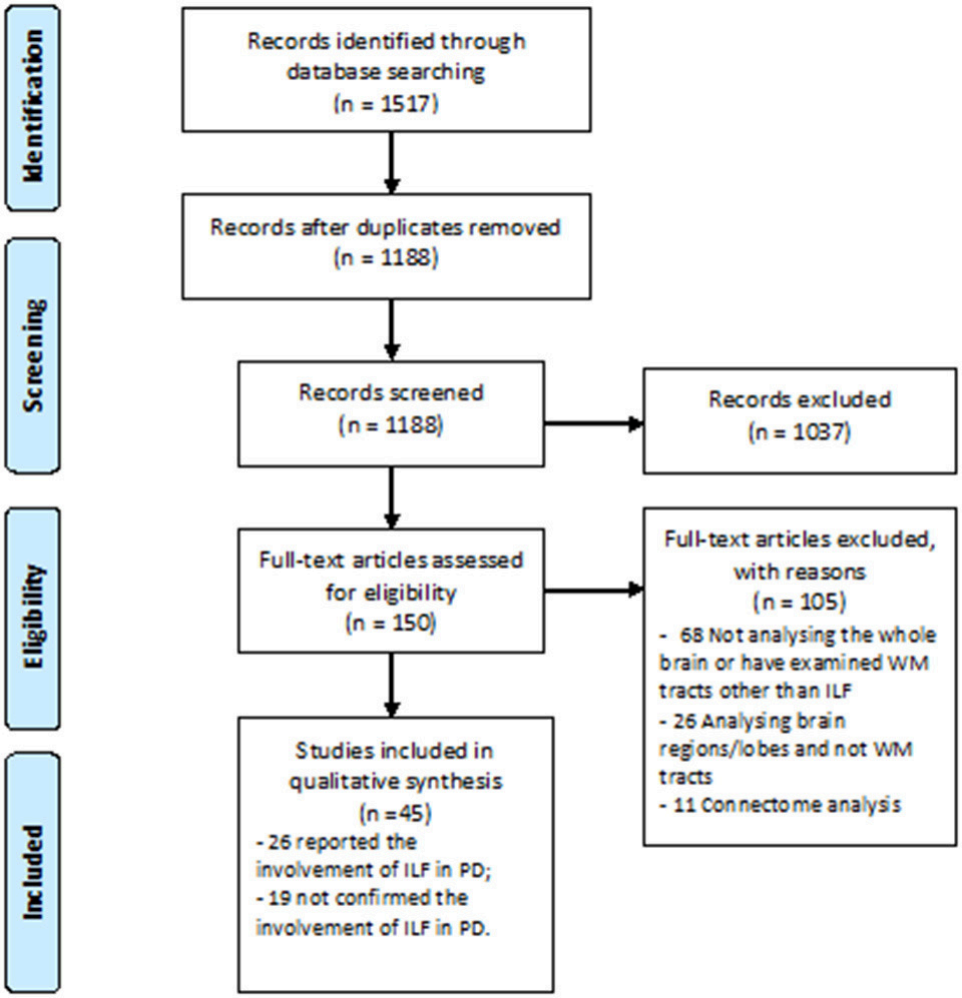
PRISMA Flow Diagram of study selection.

**Figure 2:**
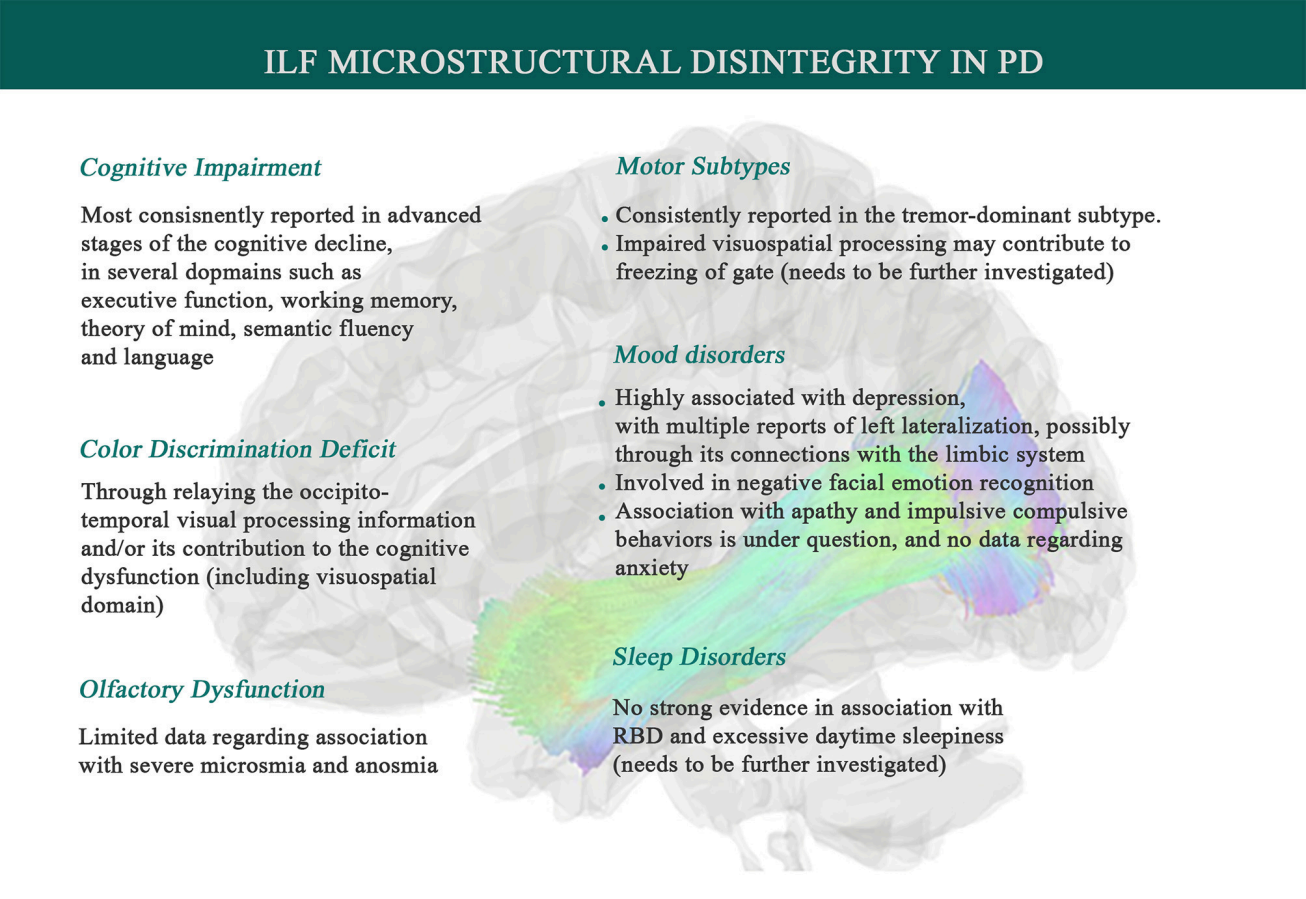
Associations between Inferior Longitudinal Fasciculus microstructural changes and Parkinson's disease symptomatology. Data suggests that systemic inflammation can lead to ILF disintegrity, exhibited by higher mean diffusivity (MD) and lower fractional anisotropy (FA), and thereby contributes to PD symptoms such as a range of cognitive impairments, depression, olfaction dysfunction, facial emotion recognition deficit, color discrimination deficit, and tremor genesis. ILF also has been inconsistently shown to play a role in sleep disorders (like RBD), impulsive compulsive behaviors, and freezing of gate, which needs to be further investigated.

A great number of studies concerning white WM changes in PD patients were excluded due to ROI or tractography analysis method which investigated other structures than ILF (Figure 1). Using Prisma guideline, we found 26 studies which have reported ILF microstructural changes in PD (Table 1). Twenty-five studies have focused on the motor or non-motor symptoms of PD (Figure 2). One study has reported a correlation between ILF microstructure and systemic inflammation in PD (64). We also found nineteen studies which despite whole brain survey, did not report any ILF involvement and are discussed as well (Table 2). Studies from all continents were included, although most studies were conducted in Europe, North America, and Asia.

**Table 1:** An overview of the literature regarding studies with significant microstructural changes of inferior longitudinal fasciculus in association with PD symptoms.

**Study**	**Groups studied**	**Number of participants (Males)**	**Mean age ± SD (years)**	**Disease duration (mean years ± SD)**	**Field strength (Tesla)**	**b-value (s/mm^2^)**	**Analysis method**	**Symptom or condition**	**ILF FA/QA comparison/correlation**	**ILF MD, AD or RD comparison/correlation**	**Methodological assessment**
Hattori et al. (39)	PD-NC PD-MCI PDD HC	32 (12) 28 (16) 25 (13) 40 (18)	75.9 ± 2 76.3 ± 6.4 80 ± 4.5 76.9 ± 4.9	5.8 ± 4.6 6.3 ± 4.3 7.8 ± 6.2	1.5	1,000	TBSS	Cognitive decline	PDD, PD-MCI < HC PDD < PD-NC Positive correlation with MMSE scores in PD (Bilateral ILF)	Not investigated	MMSE, Clinical Dementia Rating
Melzer et al. (40)	PD-NC PD-MCI PDD HC	63 (43) 28 (18) 18 (16) 32 (22)	64.0 ± 9.2 71.0 ± 7.3 73.7 ± 6.5 70.1 ± 9.0	3.7 ± 3.2 5.8 ± 5.1 12.3 ± 8.6	3	1,000	TBSS	Cognitive decline	PDD, PD-MCI < HC Positive correlation with attention, working memory, and processing speed score in posterior WM (Bilateral ILF)	PDD> PD-MCI > HC (MD, Bilateral ILF)	MDS Task Force criteria for diagnosis of dementia, Comprehensive neuropsychological testing
Chen et al. (41)	PD-NC PDD HC	19 (9) 11 (5) 21 (10)	59.47 ± 8.771 64.09 ± 11.35 61.10 ± 8.336	3.21 ± 1.960 3.64 ± 2.693	3	Not specified	ROI	Cognitive decline	–	PDD > PD-NC Negative correlation with MoCA scores in PD (MD, Left ILF)	comprehensive neuropsychological testing, MDS Task Force criteria for dementia classification, MMSE, MoCA
Koshimori et al. (42)	PD HC	26(13) 15 (4)	70.5 ± 5.6 67.13 ± 5.1	6.7 ± 4.2	3	1,000	TBSS	Cognitive decline	–	PD> HC Negative correlation with global composite z and executive composite z in PD (MD, Bilateral ILF)	extended neuropsychological test battery, MoCA
Duncan et al. (43)	PD HC	125 (85) 50 (29)	66.0 6 ± 10.5 65.86± 8.0	6.15 ± 4.66 months	3	1,000	TBSS	Cognitive decline	–	PD > HC PD-impaired semantic fluency> PD-NC, HC associated with performance on the semantic fluency and Tower of London tasks in PD (MD, Bilateral ILF)	Cognitive Drug Research battery, Neuropsychological Test Automated Battery, MoCA, MMSE
Gallagher et al. (44)	PD HC	15 (12) 15 (9)	62.7 ± 10 60.3 ± 6.5	5.6 ± 5	3	Not specified	TBSS	Executive dysfunction	PD < HC Positive correlation with executive composite scores and Stroop interference scores in PD (Bilateral ILF)	PD > HC Negative correlation with Stroop interference scores in PD (MD, Bilateral ILF)	Neuropsychological test battery
Díez-Cirarda et al. (45)	PD HC	37 (22) 15 (11)	67.97 ± 6.17 65.07 ± 7.01	6.96 ± 5.61	3	1,000	TBSS	Theory of mind deficit	–	Correlated with ToM deficit in PD (MD, RD, Left ILF)	Neuropsychological test battery
Lucas-Jimenez et al. (46)	PD HC	37 (22) 16 (12)	67.97 ± 6.18 65.13 ± 6.78	6.96 ± 5.61	3	1,000	TBSS/ROI	Cognitive decline, DMN	PD < HC (Bilateral ILF) Positive correlation with DMN functional connectivity (Right ILF)	–	Neuropsychological test battery
Theilmann et al. (47)	PD HC	25 (14) 26 (13)	68.0 ± 8.9 65.9 ± 8.4	7.2 ± 4.8	1.5	1,000	TBSS	Cognitive decline	PD < HC (Right ILF) No correlation with cognitive scores	PD > HC (MD, Right ILF; RD, Bilateral ILF)	MMSE, Judgment of Line Orientation Test (Visuospatial functioning), Digits Span Forward and Backward from the WAIS III (verbal working memory), Delis–Kaplan Executive Function System (verbal fluency), Trail Making Test (cognitive flexibility), Stroop Interference (inhibitory control)
Zheng et al. (48)	PD	15 (11)	62.2 ± 9.6	9.5 ± 6.0	3	1,000	ROI	Cognitive decline	Negative correlation with language and attention (Left sagittal stratum including ILF)	Positive correlation with executive function and attention (MD, Left sagittal stratum including ILF), and language (MD, Bilateral sagittal stratum including ILF)	Comprehensive neuropsychological testing
Kamagata et al. (49)	PD PDD HC	20 (8) 20 (10) 20 (10)	71.6 ± 4.3 71.7 ± 5.3 72.7 ± 3.3	94.0 ± 53.4 months 146.6 ± 91.0 months	3	1,000	TBSS	Cognitive decline	PDD < HC (Bilateral ILF) No correlation with MMSE scores	PDD > HC (Bilateral ILF)	MMSE (Japanese version)
Sobhani et al. (50)	PD-Anosmia PD-Severe microsmia PD-Moderate microsmia PD-Mild microsmia PD-Normal olfaction HC	18 (11) 26 (14) 17 (17) 12 (8) 12 (5) 36 (22)	58.7 ± 7.5 59.3 ± 9 57.4 ± 9.9 54.7 ± 8.8 53.4 ± 7.8 60.1 ± 10.6	6.9 ± 9 6.6 ± 5.9 7.45 ± 7.8 4.45 ± 1.6 6.27 ± 4.6	3	1,000	Connectometry	Olfactory dysfunction	PD-Anosmia < PD-Normal Olfaction PD-Severe microsmia < PD-Mild microsmia (QA, Left ILF)	Not investigated	Pennsylvania Smell Identification Test
Ford et al. (51)	PD-RBD PD-non-RBD	46 (36) 78 (48)	66.4 ± 9.9 65.8 ±10.9	6.5 ± 5.1 months 6.0 ± 4.4 months	3	1,000	TBSS	RBD	PD-RBD < PD-non-RBD (Bilateral ILF) (only significant in the uncorrected analysis)	PD-RBD > PD-non-RBD (Bilateral ILF)	question 1 on the Mayo Sleep Questionnaire
Huang et al. (52)	dPD ndPD	15 (9) 15 (9)	54.5 ± 12.2 54.8 ± 10.1	5.3 ± 4.8 4.2 ± 4.0	3	1,000	TBSS	Depression	dPD < ndPD (Bilateral ILF) Left deep temporal cortex negatively correlated with severity of depression	–	HDRS
Wu et al. (53)	dPD ndPD	31 (18) 37 (23)	58.8 ± 8.67 59.1 ± 11.4	3.23 ± 3.04 2.40 ± 2.53	3	1,000	ROI (PANDA)	Depression	dPD < ndPD (Left sagittal stratum, including ILF, no correlation with HDRS scores)	Not investigated	DSM IV criteria by an experienced psychiatrist, HDRS
Ghazi Sherbaf et al. (54)	dPD ndPD	14 (11) 18 (11)	58.28 ± 8.37 59.42 ± 11.3	7.5 ± 7.5 8.5 ± 7.5	3	1,000	Connectometry	Depression	dPD < ndPD (QA, Left ILF)	Not investigated	Geriatric depression scale
Ansari et al. (55)	dPD ndPD	40 (21) 19 (10)	57.28 ± 7.9 57.5 ± 9.38	Not specified (early drug-naïve)	3	1,000	Connectometry	Depression	dPD < ndPD (QA, Bilateral ILF)	Not investigated	Geriatric depression scale
Imperiale et al. (56)	PD-ICB PD-non-ICB HC	35 (30) 50 (36) 50 (35)	62.0 ± 10.4 61.5 ± 8.9 59.0 ± 12.4	9.5 ± 5.2 9.0 ± 6.1	1.5	Not specified	probabilistic tractography	Impulsive compulsive behaviors	–	PD>HC (AD, Right ILF)	Diagnosis: current criteria based on comprehensive clinical interview by an expert neurologist and a trained neuropsychologist, QUIP Severity: QUIP rating scale
Mojtahed Zadeh et al. (57)	PD-ICD PD-non-ICD HC	21 (14) 68 (44) 23 (12)	57.7 ± 9.8 59.1 ± 9.5 58.3 ± 10.5	10.4 ± 10.5 5.8 ± 5.3	3	1,000	Connectometry	Impulsive compulsive disorder	PD-ICD < HC (QA, Bilateral ILF) PD-non-ICD < HC (QA, Left ILF)	Not investigated	Questionnaire for Impulsive-Compulsive Disorders
Bertrand et al. (58)	PD HC	26 (17) 7 (3)	64.08 ± 8.57 70.43 ± 9.92	5.12 ± 3.24	3	700	TBSS	Color discrimination deficit	–	Positive correlation with FM 100 performance (MD, small part of Right ILF) higher MD and RD in small part of Right ILF in PD with poor performance on the FM-100	Farnsworth-Munsell 100 hue test
Baggio et al. (59)	PD HC	39 (27) 23 (12)	63.5 ± 11.4 61.0 ± 9.7	5.6 ± 3.8	3	1,000	TBSS/ ROI	Facial emotion recognition	Negative correlation with sadness recognition in PD (Left ILF; no difference in between-group comparison)	Not investigated	Ekman 60 test (sadness sub-scores)
Li et al. (60)	PD HC	31 (16) 22 (12)	60.5 ± 9.3 59.7 ± 8.6	Not specified (early stages)	3	1,200	TBSS	Motor severity in early PD	PD < HC (Left sagittal layer including ILF, no WM tract correlated with motor severity)	Not investigated	UPDRS (total, motor)
Pietracupa et al. (61)	PD-FOG PD-non-FOG HC	21 (16) 16 (13) 19	66.3 ± 10.72 69,7 ± 11.1 66.74 ± 7.68	11 ± 6.3 9.5 ± 6.2	3	1,000	TRACULA	Freezing of gate	– No correlation between DTI metrics in ILF and disease duration, H&Y, UPDRS III, MMSE, Frontal Assessment Battery, FOG-Q and Hamilton Depression Scale	PD-FOG > HC (MD, Right ILF)	Diagnosis: Timed get Up and Go test Severity: FOG Questionnaire
Wen et al. (62)	PD-TD PD-PIGD HC	52 (32) 13 (10) 61 (41)	60.46 ± 9.57 66.66 ± 10.17 60.19 ± 10.80	7.52 ± 8.00 months 6.54 ± 6.78 months	3	1,000	TBSS	Motor Subtypes (TD/PIGD)	TD > PIGD, HC Negative correlation with motor severity in PD-PIGD (Bilateral ILF)	TD < PIGD, HC (RD, Bilateral ILF) Positive correlation with motor severity in PD-PIGD (RD, AD, Bilateral ILF) No correlation with MoCA and GDS scores	tremor score/PIGD score based on UPDRS II and III
Luo et al. (63)	PD-TD PD-non-TD HC	30 (16) 30 (15) 26 (13)	53.42 ± 10.22 52.55 ± 7.33 54.46 ± 8.32	2.0 ± 1.71 2.35 ± 1.78	3	1,000	TBSS	Tremor	–	PD-TD > PD-non-TD, HC Positive correlation with resting tremor score (MD, AD, Bilateral ILF)	UPDRS (motor)
Chiang et al. (64)	PD HC	66 (23) 67 (29)	58.1 ± 8.7 56.8 ± 9.8	3.856 ± 3.588	3	1,000	ROI	Systemic inflammation	PD < HC (Bilateral ILF) Negative correlation with granulocyte LFA-1, granulocyte apoptosis (Left ILF) and lymphocyte apoptosis (Right ILF)	PD > HC (MD, Left ILF; RD, Right ILF) Positive correlation with granulocyte LFA-1, granulocyte apoptosis (RD, Left ILF) Positive correlation with P-selectin (MD, Left ILF)	Leukocyte apoptosis, adhesion molecules

*PD, Parkinson's disease; HC, healthy controls; FA, fractional anisotropy; MD, mean diffusivity; AD, axial diffusivity; RD, radial diffusivity; TBSS, tract-based spatial statistics; ROI, region of interest; VBA, voxel-based analysis; PANDA, pipeline for analyzing brain diffusion images; TRACULA, Tracts constrained by underlying anatomy; PDD, PD with dementia; PD-MCI, PD with mild cognitive impairment; PD-NC, PD with normal cognition; RBD, REM sleep behavior disorder; dPD, depressed PD; ndPD, non-depressed PD; ICB, impulsive compulsive behaviors; FOG, freezing of gait; TD, tremor dominant; PIGD, postural instability and gait difficulty; MMSE, mini-mental state examination; MoCA, Montreal cognitive assessment; ToM, theory of mind; HDRS, Hamilton depression rating scale; DSM-IV, diagnostic and statistical manual of mental disorders fourth edition; QUIP, questionnaire for impulsive–compulsive disorders in Parkinson's disease; UPDRS, unified Parkinson's disease rating scale; MDS, Movement Disorders Society; DMN, default mode network; LFA-1, lymphocyte function-associated antigen-1*.

In most of the studies, PD was diagnosed according to the UK Brain Bank criteria, and patients had primary Parkinson's disease. Hoehn and Yahr staging scale (H&Y scale) and the Unified Parkinson's Disease Rating Scale (UPDRS) were used as measures of disease severity and were significantly different between PD and healthy controls (HC) (84). Magnetic resonance images in all studies were acquired in either the field strength of 3.0 Tesla or 1.5 Tesla. Diffusion tensor imaging acquisitions were based on a diffusion spin echo sequence; features of this stage varied slightly in different studies and b-values are provided in Tables 1, 2.

**Table 2:** An overview of the literature regarding studies with no reports of significant microstructural changes of inferior longitudinal fasciculus and in association with different variables in Parkinson's disease.

**Study**	**Groups studies**	**Number of participants (Males)**	**Mean age ± SD (years)**	**Disease duration (mean years ± SD)**	**Field strength (Tesla)**	**b-value (s/mm^2^)**	**Analysis method**	**Symptom or condition**	**Methodological assessment**
Agosta et al. (65)	PD-NC PD-MCI HC	13 (6) 30 (123) 33 (17)	63.9 ± 7.1 66.6 ± 8.2 64 ± 7.3	10.0 ± 7.1 8.7 ± 5.9	1.5	1,000	TBSS	Cognitive decline: PD-MCI < PD-NC: all domains except for the language subtest of the Addenbrooke's Cognitive Examination (between-groups comparison, correlation with cognition scores)	MMSE, Addenbrooke's Cognitive Examination Revised, Frontal Assessment Battery, Rey Auditory Verbal Learning Test for memory, attention, visuospatial abilities, language, verbal fluency and executive functions
Rektor et al. (66)	PD-NC HC	20 (11) 21 (8)	61.9 ± 7.63 57.9 ± 7.24	5.6 ± 5 years	3	1,000	TBSS	Intact cognition (between-groups comparison)	Mattis Dementia Rating Scale, Tower of London, Stroop Test, Rey-Osterrieth Complex Figure Test, Wechsler Memory Scale III, Wechsler Adult Intelligence Scale-, Third Edition, Verbal Fluency Test for visuospatial, memory, attention, language, and executive function
Price et al. (67)	PD Non-PD	40 (32) 40 (33)	67.80 ± 5.44 68.18 ± 4.64	7.50 ± 5.15	3	1,000	TBSS	Cognitive decline: PD < non-PD: processing speed, working memory, Reasoning, visual perceptual/spatial, and memory composite scores (between-groups comparison, correlation with cognition scores)	A battery of neuropsychiatric tests
Ansari et al. (68)	PD-RBD PD-non-RBD	23 (18) 31 (20)	59.43 ± 10.97 60.64 ± 8.65	7.95 ± 8.76 months 7.32 ± 8.19 months	3	1,000	Connectometry	RBD (between-groups comparison)	RBD screening questionnaire
Lim et al. (69)	PD-RBD PD-non-RBD HC	24 (12) 14 (8) 25 (13)	69.8 ± 6.4 69.7 ± 7.2 68.5 ± 6.6	6.2 ± 2.9 4.4 ± 3.7	3	800	TBSS	RBD (between-groups comparison)	RBD screening questionnaire, confirmed with videopolysomnography
Chondrogiorgi et al. (70)	PD-EDS PD-non-EDS	24 (14) 14 (10)	65.8 65.7	8.2 5.7	1.5	700	TBSS	Excessive Daytime sleepiness (between-groups comparison)	Epworth Sleepiness Scale
Zhang et al. (71)	PD-apathy PD-non-apathy	18 (11) 21 (14)	62.28 ± 13.02 60.24 ± 10.32	4.06 ± 2.34 3.74 ± 2.50	3	1,000	PANDA	Apathy (between-groups comparison and correlation with apathy scores)	Diagnosis: criteria for apathy Severity: Lille Apathy Rating Scale
Canu et al. (72)	PD-punding PD-non-ICB HC	21 (18) 28 (19) 28 (19)	63.8 ± 8.8 63.6 ± 6.5 61.9 ± 8.3	9.4 ± 5.4 9.7 ± 5.4	1.5	1,000	tractography	Punding	Diagnosis: Clinical interview Severity: Punding Rating Scale
Yoo et al. (73)	PD-ICD PD-non-ICD HC	10 (7) 9 (6) 18 (10)	54.5 ± 6.2 59.6 ± 8.6 54.4 ± 6.5	10.2 ± 7.3 10.6 ± 3.9	3	1,000	TBSS	Impulsive compulsive disorder (between-groups comparison)	Diagnosis based on one or more behaviors that met the criteria of the American Psychiatric Association (Diagnostic and Statistical Manual of Mental Disorders, Fourth Edition, Text Revision, 1994)
Wang et al. (74)	PD-FOG PD-non-FOG HC	14 (9) 16 (10) 16 (8)	72.36 ± 6.15 68.88 ± 6.0 68.56 ± 2.56	3.29 ± 1.65 3.70 ± 2.94	3	1,000	PANDA, TBSS	Freezing of gait (between-groups comparison and correlation with FOG severity scores)	Diagnosis: UPDRS III Severity: Freezing of gait questionnaire
Vercruysse et al. (75)	PD-FOG PD-non-FOG HC	11 (8) 15 (11) 15 (11)	68.6 ± 8.7 67.6 ± 5.6 68.1 ± 6.5	9.5 ± 3.7 7.6 ± 5.3	3	7,000, 1,000, 2,800	TBSS	Freezing of gait (between-groups comparison and correlation with FOG severity scores)	A score of 1 or higher on the New FOG Questionnaire
Canu et al. (76)	PD-FOG HC	23 (7) 35 (15)	66.9 ± 8.0 67.7 ± 7.6	PD-FOG = at least 5 years	1.5, 3	900	TBSS	Freezing of gate (between-groups comparison and correlation with FOG severity scores)	Diagnosis: A score of 1 or higher on the New FOG Questionnaire Severity: FOG Questionnaire
Lee et al. (77)	PD	12 (2)	60.3 ± 9.1	1.2 ± 0.8	3	600	TBSS	sequence effect (Correlation)	Pentagon drawing test
Gu et al. (78)	PD-PIGD PD-non-PIGD	12 (8) 12 (7)	55.7 ± 8.1 56.0 ± 8.4	3.2 ± 3.1 8.0 ± 3.8	3	1,000	VBA	Postural instability and gait difficulty (between-groups comparison)	Mean tremor score/mean PIGD score
Haghshomar et al. (79)	PD	81 (49)	57.5 ± 8.5	6.9 ± 9	3	1,000	Connectometry	Peripheral inflammation (Correlation)	Neutrophil to lymphocyte ratio
Zhang et al. (80)	PD HC	122 (79) 50 (32)	60.5 ± 9 60.6 ± 11	Not specified	3	1,000	ROI	Longitudinal changes in regional DTI and correlation with other markers	UPDRS, MoCa, Putaminal DAT and CSF biomarkers (α-synuclein, β-amyloid, total and phosphorylated tau proteins)
Kim et al. (81)	PD HC	64 (22) 64 (22)	62.9 ± 9 63.0 ± 8.9	5.3 ± 5.4	3	800	TBSS	microstructural deficits in PD (between-groups comparison)	-
Agosta et al. (82)	PD-GBA PD-non-carrier HC	15 (9) 14 (8) 16 (9)	64 ± 8 64 ± 7 64 ± 8	10 ± 6 11 ± 6	1.5	1,000	TBSS	Glucocerebrosidase gene mutations (between-groups comparison and correlation of DTI metrics with cognitive scores)	-
Kikuchi et al. (83)	PD-MIBG-H PD-MIBG-L	12 (5) 12 (5)	66.8 ± 4.9 67.4 ± 6.1	1 ± 1.3 2 ± 1.9	3	800	TBSS	MIBG uptake	I-MIBG scintigraphy

*PD, Parkinson's disease; HC, healthy controls; FA, fractional anisotropy; MD, mean diffusivity; TBSS, tract-based spatial statistics; ROI, region of interest; VBA, voxel- based analyses; PANDA, pipeline for analyzing brain diffusion images; PDD, PD with dementia; PD-MCI, PD with mild cognitive impairment; PD-NC, PD with normal cognition; RBD, REM sleep behavior disorder; ESS, excessive daytime sleepiness; ICB, impulsive compulsive behaviors; ICD, impulsive compulsive disorder; FOG, freezing of gait; MMSE, mini-mental state examination; MoCA, Montreal cognitive assessment; UPDRS, unified Parkinson's disease rating scale; GBA, glucocerebrosidase gene mutations; MIBG, I-metaiodobenzylguanidine; MIBG-H, high MIBG uptake; MIBG-L, low MIBG uptake*.

### ILF and non-motor symptoms

#### Cognitive impairment

Cognitive impairment is a prominent feature of PD, and its investigation is a topic of growing interest (85, 86). Especially, executive dysfunction is one of the key aspects of cognitive impairments in these patients (87). Mild cognitive abnormalities increase the risk of developing dementia and thus, having a conception of their neural substrates can help develop more potent and targeted disease modifications to prevent dementia (88).

Frontal-subcortical networks participate in working memory, attention, cognitive flexibility, and impulse control functions. In PD patients, cholinergic loss, focal cerebral and subcortical volume loss (89), and loss of dopaminergic input to the striatum modulate these networks, and thus, these functions are not performed properly. Moreover, it has been consistently shown that the temporal and occipital regions (like temporal pole, lingual gyrus, fusiform gyrus, and cuneus) are extensively damaged in PD with mild cognitive impairment (PD-MCI) compared to cognitively normal PD (PD-NC) (44). In addition, PD-MCI patients converting to dementia (PDD) have shown progressive cortical thinning in these regions (90). ILF is a critical pathway connecting inferior occipital gyrus to the temporal pole passing through fusiform gyrus and visual word form area (29, 30). Thus, it can be speculated that ILF damage may be one of the pathological substrates of specific domains of cognitive deficit in PD. Studies which have investigated the associations between microstructural changes of ILF and cognitive status in PD patients are reviewed in the following paragraphs.

Hattori et al. used TBSS analysis to investigate FA changes of WM in magnetic resonance images of subgroups of PD patients with different cognitive status (39). The comparison was made between patients and HC and also between PD subtypes. PDD patients were significantly older than PD-MCI or PD-NC patients. Compared to HC, lower FA values of ILF were detectable in PD-MCI and PDD, but not in PD-NC group. Moreover, positive correlations between mini-mental state examination (MMSE) scores in patients with PD and FA values of some WM tracts including ILF were reported.

Similarly, Melzer et al. investigated the differences of WM MD and FA between PDD, PD-MCI, PD-NC and HC groups using TBSS analysis of DTI data (40). Movement Disorder Society (MDS) Task criteria was the basis of dementia diagnosis. They discovered lower FA and higher MD values in bilateral ILF in PDD and PD-MCI groups compared to both PD-NC and HC groups.

Chen et al. used DTI and functional MRI (fMRI) to seek the probable differences between PDD, PD-NC, and HC groups in terms of structural WM integrity and functional connectivity (41). This study showed higher MD values of the left ILF in PDD compared to PD-NC but not compared to HC. In addition, a negative correlation was observed between MoCA scores and MD values of the left ILF.

Koshimori et al. addressed WM alterations using TBSS, in order to clarify structural abnormalities and their relationship with cognitive performance in non-demented PD patients (42). The neuropsychological assessment showed poorer performance on MoCA, global composite z, executive composite z, visual-verbal test and judgment of line orientation among PD subjects compared to HC. They observed WM alterations like significantly higher MD values in PD patients compared to HCs in widespread tracts, including bilateral ILF, with negative correlation with global function and executive function.

Duncan et al. performed a study to seek the evidence of alterations in regional GM volume and integrity of the principal WM tracts in newly diagnosed PD patients (with disease duration of less than a year) (43). Patients were assessed “on medication”. PD subjects had small but significant reductions in performance on tests of global cognition and all domain-specific cognitive tests, compared to controls after Bonferroni's correction for multiple testing. They hypothesized that changes in MD would be detectable before the observation of any reduced FA and significant GM loss; as MD is a more sensitive marker of WM damage. They found, averaged across the entire WM, PD subjects had greater MD than controls. Investigation with TBSS showed MD increases in several WM tracts including the ILF in PD patients. Significantly higher MD value was also observed in PD subjects with impaired semantic fluency, relative to PD-NC and controls assessed with semantic fluency and Tower of London tasks. However, there was no difference in total FA between control and PD subject groups, nor was there any significant association between FA values and cognitive performance.

Gallagher et al. hypothesized impaired microstrutural integrity within frontal-subcortical WM tracts would be related to executive dysfunction in PD patients (44). Their research by using TBSS analysis showed that lower FA and higher MD in PD group were present in several WM tracts, including ILF. It was notable that most regions of the brain showed higher MD in PD, compared to less widespread changes in FA. Higher FA index (but not lower MD index) was related to higher executive function (composite Z scores) in patients with PD. Both higher FA and lower MD indices were related to disrupted inhibitory control (higher Stroop Interference scores) in PD.

Theory of mind (ToM) is defined as the ability to reason and judge about one's or other's mental status. Due to the presence of delusions in demented patients, ToM deficit is frequent in PD patients especially those with dementia and executive dysfunction (91, 92). Díez-Cirarda et al. aimed to find the neural correlates of ToM in PD patients and its relations with working memory and executive function, using TBSS method (45). Controlling for executive function, ToM deficit in PD patients was correlated with MD and RD values (but not FA, and AD) in some brain regions, including the left ILF.

Lucas-Jimenez at al. assessed connectivity in the default mode network (DMN) with resting-state fMRI and also obtained DTI images that were processed by TBSS, and carried a battery of cognitive tests (46). They found lower DMN functional connectivity in PD which was accompanied by deficits in verbal and visual memory tasks and visual ability performance. In addition, it was correlated with reduced FA value of the right ILF in PD patients.

Theilmann et al. applied TBSS method to find the neural correlates of cognitive function in several domains of executive functioning (working memory, verbal fluency, cognitive flexibility, inhibitory control) and visuospatial ability in a group of non-demented PD vs. HC (47). TBSS analysis revealed reduced FA and increased MD in the right ILF and higher RD in bilateral ILF with no AD changes in between-group comparison. Through ROI analysis, they reported several brain regions, but not ILF fibers, with significantly reduced FA in PD compared with HC, each correlated with specific components of cognitive function. Especially and in contrast to their hypothesis, they did not find any contribution of posterior cortical areas in visuospatial processing measured by Judgment of Line Orientation.

Zheng et al. designed their DTI study to search into the neural substrates of five distinct cognitive domains, namely executive function, short-term and long-term memory, visuospatial skills, language, and attention in PD patients without using any control group (48). They assessed FA and MD values using ROI analysis and confirmed the results by applying VBA. Due to using ROI method, the resolution was not enough to discriminate between ILF and other parts of the sagittal stratum. Executive dysfunction and language were correlated with higher anisotropy in the left ILF, and attention deficits showed a pattern of bilateral ILF anisotropy reduction. The visuospatial skill was the only cognitive domain without any association with diffusivity measures of WM tracts.

Kamagata et al. investigated the correlation between cognitive decline and WM deficits in PDD and PD without dementia by applying TBSS to the DTI data (49). ILF was not among multiple major WM tracts that showed significant FA reductions in PDD compared to PD patients. However, PDD showed loss of integrity in terms of lower FA and higher MD in widespread WM tracts, including ILF, compared to HC. The authors have not performed between-group comparison between PD without dementia and HC. Furthermore, there was no correlation between diffusivity metrics in the ILF and MMSE scores in all PD patients. Despite these interesting results regarding the contribution of loss of ILF integrity in association with cognitive impairment, there are several reports that have not confirmed this debate. Agosta et al. in a query to identify patterns of microstructural alterations in PD-MCI and PD-NC patients, applied TBSS analysis (65). PD-NC did not have any changes in WM compared to HC. PD-MCI did show disruption of various WM tracts in comparison with PD-NC and HCs. However, they did not find ILF disruptions, even though, they compared PD-MCI and cognitively intact patients with relatively long disease duration. This can be attributed to the fact that they used 1.5 T strength field, which is less accurate than 3 T studies in diagnosing the structural abnormalities (93).

In a group of PD patients with normal cognition tested for attention, executive function, working memory, and visuospatial and language domains, Rektor et al. recently have tried to assess WM disruptions in PD patients compared to HC using TBSS (66). ILF did not show any differed integrity between these groups. However, it is important to note that PD patients in this study were cognitively unimpaired, which leads us to infer that ILF involvement might happen later in the course of the disease with possible upcoming deterioration in the cognitive status, and intact cognition requires a normal ILF.

In another study by Price et al. WM alterations in relation to cognitive decline in non-demented PD patients with deficits in working memory, abstract reasoning and also visuoperceptual abilities were assessed using TBSS (67). They found mostly frontostriatal deficits, the main and primary areas responsible for cognition, and ILF was not among the reported fibers. It is possible that if PD patients with dementia were investigated in these studies, or disease duration was more prolonged they would manifest more diffuse WM alterations including ILF.

#### Summary

Research in this area is quite novel and demands a lot more evidence. Taken together, these results indicate that loss of microstructural WM integrity alterations in PD associates with the progression of cognitive dysfunction. These studies indicate significant microstructural differences between groups of PD patients with various levels of cognitive decline in multiple domains. Despite some discrepancies in the current literature, interestingly, most of the studies did not show a difference between PD-NC and HC groups in ILF integrity. This observation may imply that cognitive dysfunction, which occurs late in the disease course, is dependent on the progressive microstructural changes in specific WM tracts such as ILF, and in those with normal cognition such changes may not yet be readily etected. It is somehow supported by the consistent finding of altered ILF in PDD among existing studies. Moreover, ILF microstructure was correlated with cognitive test scores and especially in relation to executive function, language, attention, working memory and semantic fluency. However, to our surprise, there are no reports of ILF disruption regarding the visual-related cognitive deficit, and it was only indirectly captured by the fMRI study by Lucas-Jimenez et al. (46).

Of note, these results show that MD changes may happen earlier in the disease course and may be a more sensitive marker of WM damage than FA changes; In fact, more extensive WM damage represented by FA changes are more robustly associated with cognitive dysfunction, while higher MD values may alarm the deterioration of cognitive performance before observing reductions in FA. This notion should be addressed in future research.

Despite these interesting results, the weight and importance of changes in ILF microstructure regarding the cognitive decline in PD compared to the whole brain are not clear. Segmentation of ILF specifically may help us understand the role of different portions of this long association fiber. Lastly, for further investigation, we propose to assess patients with impaired visual tasks, in order to better clarify whether the key function of ILF has any role in related cognitive malperformance in PD.

#### Olfaction dysfunction

Olfactory dysfunction is an established PD prodromal marker. It is present in about 90% of PD patients in early stages of the disease and can be later followed by motor symptoms (94). The mechanisms and pathways resulting in an impaired sense of smell are yet to be known. Glomeruli, functional units of the olfactory bulb, have reduced size in PD patients. In addition, olfactory dysfunction is related to lower numbers of neurons in the locus coeruleus, and the raphe nuclei (95). Gyrus rectus and primary olfactory area are shown to have reduced FA values in PD patients with severe microsmia and anosmia compared to PD patients with mild/moderate or no olfactory dysfunction, assessed by Pennsylvania Smell Identification Test (UPSIT) scores (96). Sobhani et al. (50), conducted DTI connectometry in PD patients with five different levels of olfaction dysfunction based on UPSIT scores and healthy controls. Only patients with anosmia and severe microsmia had a robust decrease in the connectivity of the left ILF. Anatomically, ILF is not related to the rectus gyrus or other olfaction areas. It must be further investigated how the progression of microsmia is associated with ILF disruptions, in case this limited data is the capture of real involvement of ILF in olfaction dysfunction in PD.

#### Sleep disorders

Rapid eye movement sleep behavior disorder (RBD) is a parasomnia, characterized by lack of muscle atonia during rapid eye movement (REM) sleep (97, 98), and is frequently observed as a prodromal symptom in Parkinson's disease and other alpha-synucleinopathies (99).

Ford et al. designed a study to make a comparison between early-diagnosed and non-demented PD patients with and without RBD assessed by Mayo Sleep Questionnaire. MRI voxel-based morphometry and TBSS analyses indicated that patients with RBD have greater cerebral atrophy and microstructural WM abnormalities (51). Differences in diffusivity were widespread but were mainly located posteriorly. The largest areas of increased MD in patients with RBD were in the right and left ILF. The two groups were matched in terms of age, gender, disease duration, motor subtype and depressive symptoms, but PD-RBD patients had higher excessive daytime sleepiness (EDS) scores as well. In contrast to this study, using a connectometry analysis, Ansari et al. (68) were able to find lower connectivity in some cerebral and cerebellar tracts but not ILF, comparing two groups of PD patients with and without RBD, assessed by RBD screening questionnaire (RBDSQ). This is the same for the study by Lim et al. (69), dividing PD patients based on scores of RBDSQ followed by video-polysomnographic confirmation. One might speculate that the positive results reported in the first study might be due to EDS rather than RBD. However, WM microstructural changes related with EDS was examined with TBSS by Chondrogiorgi et al. (70). The results did not show alterations in the ILF integrity.

Evidence regarding the role of ILF in sleep disorders is sparse. RBD requires activation of motor system components during REM sleep, and the number of studies which have found ILF disruption in relation to motor deficits in PD is very limited (discussed below), implying that ILF disruption may not play an important role in sleep disorders.

#### Mood disturbance

It is now well cleared that depression may evolve from neurodegenerative processes in PD and is marked as an established Parkinson's prodromal marker (100, 101). With an estimated prevalence of at least 30–40% (102), depression is the main culprit in a lower quality of life in PD patients (103), and more importantly, alarms the development of dementia and thus more advanced disease with poorer prognosis (3, 104–106).

ILF is the main component of the visual-limbic pathway that subserves emotional, learning and memory functions that are modality specific to vision (107). Clinical investigation has revealed that depressed patients suffer from a functional visual loss (108). A meta-analysis of DTI studies has also manifested the role of ILF in major depressive disorder (109). It is interesting to note that all the studies investigating the neural correlates of depression in PD (dPD) have consistently reported the ILF involvement. Comparing dPD to non-dPD patients, lower FA in the bilateral (52), or left ILF (53), is shown to be associated with depressive symptoms and its severity (52). Lower connectivity in the left ILF, comparing groups of dPD and non-dPD patients with comorbid RBD through diffusion MRI connectometry was also reported in a recent study by Ghazi Sherbaf et al. (54). Using the same method, Ansari et al. (55) searched for diffusion changes in dPD vs. non-dPD patients without comorbid RBD. dPD group showed significantly reduced connectivity in bilateral ILF.

Reports of of left lateralization in PD is also observed among other WM tracts in dPD (53) and agrees with the assumption that depression is associated with a hypoactivity in the left hemisphere (110). More interestingly, right-onset PD patients will experience more severe depressive symptoms in the course of the disease (111). Despite these interesting results, more studies on larger groups of patients are needed to verify the generalizability of this association.

Apathy, another significant mood disturbance in PD has been investigated by a few studies, and only one DTI study has tried to probe the underlying neural disruptions in relation to this symptom. However, it has shown that PD patients with apathy do not have distinguished ILF microstructure compared with patients without apathy (71). Apparently, more studies are needed to validate this result. The literature also lacks studies investigating the role of ILF in anxiety in PD.

#### Impulsive-compulsive behaviors

Long-term dopaminergic treatment in PD patients can lead to impulsive and compulsive behaviors (ICBs) (112).

Imperiale et al. performed a study to find out WM alterations associated with ICB (56). Using probabilistic tractography, they showed that right ILF had an increased AD in PD-ICB and PD-non-ICB patients compared to controls, but ILF microstructure was not significantly different between ICB and non-ICB groups. It must be added that punding, known as irrational and intense affection to an aimless repetitive activity was the most observed ICB. However, Canu et al. did not observe any microstructural changes in ILF, specifically checked in tractography, in the between-group analysis of PD patients with and without punding and HC (72). Yoo et al. also searched for WM alterations in medication-induced ICB in PD, comparing PD-ICB, PD-non-ICB and HC with DTI collected images analyzed with TBSS (73). They did not find any FA or MD alterations in the ILF of ICB subtype. A novel whole WM connectometry study has also tried to find out the neural correlates of ICB in early drug-naïve PD patients without the interference of dopaminergic drugs. Among the structures with lower connectivity comparing PD-ICB, and PD-non-ICB, ILF is not reported (57). Although, ILF had lower quantitative anisotropy (QA) comparing both subgroups of PD patients with HC. It is important to note that the observed ILF disruption comparing PD-ICB versus HC in this study, may be due to the reported higher geriatric depression scale and/or poorer olfaction function in PD-ICB. This is the same for the first mentioned study (56), with reported higher scores on the Hamilton depression rating scale in PD-ICB. Therefore, the reported associations of ILF disruption with ICB is under question, considering the fact that both of these studies have not found such difference comparing two groups of PD patients with and without ICB. Since mesocorticolimbic network serves the key role in the pathology of ICB (113), it seems that ILF, as a main component of the visual-limbic pathway (114, 115), may not be associated with impulsivity in medicated or drug-naïve PD.

#### Color discrimination deficits

Parkinson's disease (PD) is frequently accompanied by sensory anomalies like color discrimination deficit (116). Color vision processing is performed by a variety of cortical neural structures in the ventral visual pathway, namely, the primary visual areas, V4 area, and regions of the inferior temporal cortex (117). ILF extending from the occipital lobe to temporal lobe is involved in visual processing (118). Bertrand et al. used cortical thickness and DTI to assess the relationship between Farnsworth–Munsell 100 hue (FM-100 HTES) test performance and cortical and WM anomalies in patients with PD. DTI analyses revealed a positive correlation between FM-100 HTES scores and MD in a small portion of the ILF. In addition, PD patients who performed poorly on the FM-100 HTES showed higher MD and RD in this fiber (58). Performance on the FM-100 in PD was significantly different in patients with cognitive deficits. FM-100 requires intact color vision, yet several other higher cognitive processes are involved in performing this test; such as attentional, executive, and visuospatial capacities. These cognitive abilities are also affected in PD (119, 120). Thus, associations between the integrity of the ILF and color discrimination in PD may point toward the role of ILF in cognition as well as visual processing.

#### Facial emotion recognition

The ability to accurately identify emotions in others' facial expressions, a skill of great importance for normal social interaction, has been described in several studies to be impaired in PD (121). Regarding identification of which specific emotions are selectively or predominantly affected, previous studies have mostly suggested a selective deficit in identifying negative emotions (fear, anger, sadness) (122). Importantly, emotion recognition relies on numerous neural substrates; In the presence of a target (a facial emotion), the orbitofrontal cortex (OFC) is activated, and it is, on the other hand, associated with the functional connectivity between a particular region of the right OFC and bilateral visual brain regions [i.e., the inferior occipital gyrus (IOG)] (123). Therefore, OFC and IOG connections consisting of some structures like ILF are associated with facial emotion recognition. To support this, DTI studies have shown that in children with object recognition deficit (124) and also in patients with progressive prosopagnosia (isolated deficit in recognition of facial emotions) (125), ILF integrity is compromised. In PD patients, H.C. Baggio et al. have studied the relationship between the capacity to recognize specific emotions in facial expressions and gray and white matter structural parameters (59). They detected a positive correlation between FA in left ILF and sadness scores despite no significant difference in FA values of ILF between PD and HC.

### ILF and motor symptoms

Li et al. analyzed WM integrity using TBSS in early PD patients and HC. They found that ILF had reduced FA in PD. However, no diffusivity measures in any WM tract, including ILF, correlated with motor severity (60).

Freezing of gate (FOG) is a prominent motor disorder of PD characterized by a difficulty to start walking and once started, being unable to stop walking (126). Neuroimaging of FOG shows regional alterations in premotor and motor areas, parts of frontal, parietal and occipital lobes, and cerebellum significantly more disturbed in the right hemisphere (127). Pietracupa et al. studied structural changes in WM of PD patients with FOG (PD-FOG) compared with patients without FOG (PD-non-FOG) with DTI using tracts constrained by underlying anatomy (TRACULA) toolbox (61). This is a new method developed to draw probabilistic mapping algorithm with the known anatomy of the WM tracts. PD-FOG showed increased MD in the right ILF tract. In a study by Wang et al. using a TBSS method, PD-FOG patients appeared abnormal in visual temporal areas compared to PD-non-FOG, but ILF tract is not mentioned exclusively (74). Another TBSS study by Vercruysse et al. did not reveal any ILF microstructural differences comparing PD-FOG, PD-non-FOG, and HC (75). ILF was again not among the widespread changes, Canu et al. found in WM tracts comparing PD-FOG with HC (76). This study was first performed with a 3T acquisition and replicated with 1.5 T to confirm the results. Out of four studies concerning FOG related WM changes, only one study was able to find MD changes in the right ILF. ILF is involved in visuospatial processing which influences beginning of movement but is not among key regions necessary for movement.

Another cardinal motor feature of PD is bradykinesia. It is defined as slowness of movement caused by a proposed basal ganglia dysfunction (128). Sequence effect (SE), a feature of bradykinesia, is a progressive decrease in speed and accuracy of repetitive tasks (77). Lee et al. (77) studied the neural substrates of SE in de novo PD patients with TBSS analysis. They did not find ILF involvement in this regard.

There are intriguing results of ILF disruption in relation to tremor, a cardinal motor feature of PD, which shows more benign clinical course and lesser burden of non-motor symptoms compared to other motor subtypes of PD such as postural instability and gait difficulty (PIGD), with possible different underlying pathogenesis (129). Wen et al. performed DTI on de novo PD patients and HC to differentiate WM changes in PD motor subtypes (62). The TBSS analysis was used to create a map of WM regional features. Motor groups consisted of tremor dominant (TD) and PIGD. TD patients showed increased FA and decreased RD in bilateral ILF compared to HC and PIGD subtype. The authors have also reported a positive correlation between AD and RD of the ILF and motor severity only in PIGD subtype. Luo et al. tried to identify WM substrates of tremor by TBSS in three groups of PD-TD, PD non-TD, and HC (63). They found PD-TD had significantly higher MD and AD in the in ILF compared with PD-non-TD. Additionally, Gu et al. exclusively searched for diffusion abnormalities in diffusivity measures with the VBA method in PIGD and non-PIGD subtypes (78). No alterations were observed in ILF. The consistent reports of ILF disruptions in relation to tremor and not to other motor features of PD points toward the probably different underlying pathology between TD and other motor subtypes. This speculation demands further investigation.

### ILF and PD pathology

There is accumulating evidence that neuroinflammation and microglial activation (130) along with systemic inflammation (131, 132) contribute to the neurodegeneration in PD pathogenesis. Chiang et al. (64) performed a study to examine the role of systemic inflammation in WM disruption in PD using ROI analysis method via two main mechanisms: (1) increased peripheral leukocyte apoptosis as a reflection of increased leukocytes and/or microglial activation by circulating cytokines, which readily cross the blood-brain barrier (BBB) (133–135), and (2) increased expression of leukocyte adhesion molecules like macrophage antigen complex-1 (Mac-1), lymphocyte function-associated antigen-1 (LFA-1), and selectins, which can activate microglia and help peripheral leukocytes pass across the BBB (136–139). This study suggests the possible role of inflammation in demyelination and axonal damage in ILF in PD patients. In particular, they showed higher inflammatory markers as well as lower FA and higher MD and RD in the ILF in PD compared to HC and in correlation with higher granulocyte and lymphocyte apoptosis, granulocyte LFA-1 and P-selectin markers.

Neutrophil to lymphocyte ratio (NLR) is another commonly used peripheral inflammatory marker, which is shown to be increased in PD patients and in correlation with the dopaminergic demise in the striatal nuclei (140). Haghshomar et al. (79) studied the correlation of NLR measures in PD patients with their whole WM connectivity using connectometry approach and revealed several pathways in this regard. Even though disease duration was significantly higher in the later study (6.9 vs. 3.856 years), authors were not able to detect the correlation between NLR and lower connectivity in the ILF in terms of QA. Different DTI analyses and metrics and the different specificity and representativeness of the inflammation markers may explain this discrepancy between two studies. Given the presence of BBB, it can be hypothesized that it takes a more distant, and maybe distinct, pathway for peripheral inflammatory cells compared to molecules to take part in neurodegeneration and this could be the underlying reason that Haghshomar et al. despite investigating patients with higher disease duration and applying a more sensitive method of analysis, did not report any correlation between peripheral inflammation and ILF anisotropy. Furthermore, it is not known if patients in this study had any disruptions in ILF, as they did not compare PD patients to HC. In fact, not all studies have shown ILF disruptions comparing PD to HC (80–83). Considering the heterogeneity of PD symptoms and the outcoming courses between PD individuals, it is reasonable that not all PD patients will demonstrate same neural disruptions.

Although peripheral inflammation is easier to measure, it is just an initiator or the outcome of central inflammatory processes in the immune-privileged brain, and the current literature clearly lacks studies investigating the correlation of central inflammation markers with WM integrity.

Regarding other proposed PD pathological mechanisms, only a few studies have tried to investigate the correlation of related markers with the white matter integrity. In this regard, glucocerebrosidase gene mutations have not proceeded with ILF disruptions (82). It is quite a novel question whether gene mutations will lead to specific white matter disruptions. However, research until now is limited, and it requires more studies to decide which specific tracts a gene is related to. Moreover, ILF was not correlated with meta iodo benzyl guanidine (MIBG) uptake as a marker of heart/mediastinum count (83). The heart is related to the brain via the vagus nerve. Vagus nucleus is located in the medulla oblongata and has no direct anatomical connections with the ILF.

## Conclusions

Diffusion tensor imaging has been widely used to study the putative relationship between WM disruptions and PD symptoms. Accumulating evidence contribute to the notion that the microstructural integrity of ILF may be altered in PD patients and the changes in MD value seem to appear earlier than alterations in FA value. Loss of integrity in the ILF represent a clinically significant correlation with some PD symptoms, most consistently with the tremor genesis, depression, negative emotion recognition, color discrimination deficit, and cognitive decline in several domains. More studies are needed to validate the limited data regarding the role of ILF in other non-motor symptoms such as olfactory dysfunction. Studies investigating the neural underpinnings of the cognition mostly claim on the later disruption of ILF integrity with the progression of cognitive decline. However, ILF is shown to be disrupted in early stages of the disease with comorbid depressive disorders, color vision loss, and probably the olfactory dysfunction. It is clear that more evidence is needed to better clarify the casual relationship between ILF neurodegeneration and PD symptomatology and other potential markers especially in the early stages of the disease, which offer the greatest opportunity to apply neuroprotective measures and halt the neurodegenerative process.

## Author contributions

MH, and MD was involved in project conception, design, systematic review searching, data extraction/tabulation, data interpretation, manuscript composition, and editing. HSM and FG were involved in manuscript composition and editing. MS was involved in design, and manuscript editing. MHA was involved in the design, data interpretation, manuscript writing, and editing.

### Conflict of interest statement

The authors declare that the research was conducted in the absence of any commercial or financial relationships that could be construed as a potential conflict of interest.
